# To Enjoin the Texas Quarantine Authorities

**Published:** 1899-10

**Authors:** 


TO ENJOIN THE TEXAS QUARANTINE AUTHORITIES.—A WILD-GOOSE CHASE.—Representatives of the business interests of New Orleans have gone to Washington to get an injunction from the United States Supreme Court restraining State Health Officer Blunt from putting embargo on freights, etc., destined for Texas. This they hope to do, under a construction of the Interstate Commerce law. The Southern Pacific Railroad Company is no party to this movement; realizing, as the General Manager writes Dr. Blunt, that were the injunction granted, county authorities could not be enjoined from quarantining; and even were such a thing possible, the *people* would fall back on primitive but effective methods of shotguns and tearing up tracks, etc.

